# Supplementary material for: Dynalign II: common secondary structure prediction for RNA homologs with domain insertions
Source: Nucleic Acids Res. 2014 Nov 21;42(22):13939–48. doi: 10.1093/nar/gku1172 (PMC4267632; doi:10.1093/nar/gku1172)
Supplement: SUPPLEMENTARY DATA [file supp_gku1172_nar-02021-z-2014-File012.zip › manual/javadoc/comments/DrawingSourceProxy.html]

A package that holds all Java drawing classes generated by SWIG that connect the C++ back end to the Java front end.
  
  
None of the classes in this package are commented.
This is because the SWIG documentation framework is deprecated, and no changes have been made to the generated files, for the sake of clarity and simplicity.
To see the documentation of the C++ classes that back these Java classes (they have the same name as the Java classes), please see the Doxygen documentation for them.
